# Lessons Learned About Designing and Conducting Studies From HRI Experts

**DOI:** 10.3389/frobt.2021.772141

**Published:** 2022-01-28

**Authors:** Marlena R. Fraune, Iolanda Leite, Nihan Karatas, Aida Amirova, Amélie Legeleux, Anara Sandygulova, Anouk Neerincx, Gaurav Dilip Tikas, Hatice Gunes, Mayumi Mohan, Nida Itrat Abbasi, Sudhir Shenoy, Brian Scassellati, Ewart J. de Visser, Takanori Komatsu

**Affiliations:** ^1^ Intergroup Human-Robot Interaction (iHRI) Lab, Department of Psychology, New Mexico State University, Las Cruces, NM, United States; ^2^ Division of Robotics, Perception, and Learning (RPL), School of Electrical Engineering and Computer Science, KTH Royal Institute of Technology, Stockholm, Sweden; ^3^ Human-Machine Interaction (HMI) and Human Characteristics Research Division, Institutes of Innovation for Future Society, Nagoya University, Nagoya, Japan; ^4^ Department of Robotics and Mechatronics, School of Engineering and Digital Sciences, Nazarbayev University, Nur-Sultan, Kazakhstan; ^5^ Lab-STICC, University of South Brittany, CNRS UMR 6285, Brest, France; ^6^ Strategy, Innovation and Entrepreneurship Area, Institute of Management Technology, Ghaziabad, India; ^7^ Affective Intelligence and Robotics Lab, Department of Computer Science and Technology, University of Cambridge, Cambridge, United Kingdom; ^8^ Haptic Intelligence Department, Max Planck Institute for Intelligent Systems, Stuttgart, Germany; ^9^ Human-AI Technology Lab, Computer Engineering Program, University of Virginia, Charlottesville, VA, United States; ^10^ Social Robotics Lab, Department of Computer Science, Yale University, New Haven, CT, United States; ^11^ Warfighter Effectiveness Research Center, U.S. Air Force Academy, Colorado Springs, CO, United States; ^12^ Department of Frontier Media Science, School of Interdisciplinary Mathematical Science, Meiji University, Tokyo, Japan

**Keywords:** methodology, qualitative, quantitative, research, statistics, human-robot interaction, reproducibility, replication

## Abstract

The field of human-robot interaction (HRI) research is multidisciplinary and requires researchers to understand diverse fields including computer science, engineering, informatics, philosophy, psychology, and more disciplines. However, it is hard to be an expert in everything. To help HRI researchers develop methodological skills, especially in areas that are relatively new to them, we conducted a virtual workshop, Workshop Your Study Design (WYSD), at the 2021 International Conference on HRI. In this workshop, we grouped participants with mentors, who are experts in areas like real-world studies, empirical lab studies, questionnaire design, interview, participatory design, and statistics. During and after the workshop, participants discussed their proposed study methods, obtained feedback, and improved their work accordingly. In this paper, we present 1) Workshop attendees’ feedback about the workshop and 2) Lessons that the participants learned during their discussions with mentors. Participants’ responses about the workshop were positive, and future scholars who wish to run such a workshop can consider implementing their suggestions. The main contribution of this paper is the lessons learned section, where the workshop participants contributed to forming this section based on what participants discovered during the workshop. We organize lessons learned into themes of 1) Improving study design for HRI, 2) How to work with participants - especially children -, 3) Making the most of the study and robot’s limitations, and 4) How to collaborate well across fields as they were the areas of the papers submitted to the workshop. These themes include practical tips and guidelines to assist researchers to learn about fields of HRI research with which they have limited experience. We include specific examples, and researchers can adapt the tips and guidelines to their own areas to avoid some common mistakes and pitfalls in their research.

## 1 Introduction

With Human-Robot Interaction (HRI) researchers coming from diverse backgrounds in computer science, engineering, informatics, philosophy, psychology, and more disciplines, we cannot be experts in everything. Often, reviewers of HRI papers lament that some papers that are robust in one area are crippled by another (e.g., a very strong application, but a weak study design, making it impossible to draw conclusions from the data). This concern is one of the major entry barriers in the HRI community. If the authors of those HRI papers had worked with an expert in a complementary field, the paper would be exceptional.

To help solve this problem, we ran a workshop (Fraune et al., 2021) at the 2021 International Conference on Human-Robot Interaction^1^ to match (mainly early-career) researchers with experts in complementary areas. Before the workshop, mentees submitted a project they were currently designing. During the workshop, they met with mentors and received feedback to enhance their study design and interdisciplinary work.

This paper is a collaborative effort between workshop organizers, mentees, and mentors. We report the main insights from this workshop, including a survey with workshop participants’ (mentees and mentors) feedback on what to improve for the next editions of the workshop, as well as a compilation of the main lessons learned from workshop discussions about designing studies in HRI. The method we use–that is, conducting a workshop to acquire guidelines for field–has also been used successfully in human-computer interaction (Mubin et al., 2016).

The results in this paper are not a comprehensive compilation of everything one needs to know for planning and executing a successful HRI study. There are many great examples of such efforts from different perspectives. Examples include an introductory textbook on methods in HRI (Bartneck et al., 2020) a systematic review and guidelines for conducting Wizard-of-Oz HRI experiments (Riek, 2012), writings on methodology trends in the HRI community, along with practical recommendations (Baxter et al., 2016; Bethel and Murphy, 2010), and a comprehensive guide for planning, executing, analyzing and reporting hypothesis-driven HRI experiments with a special focus on quantitative methods (Hoffman and Zhao, 2020). Others have compiled insights relevant to HRI studies with specific user groups such as children (Ros et al., 2011).

Contrasting with previous literature, our main goal with this paper is: 1) To provide an overview of what types of improvements are more often suggested for studies, and 2) To focus on the more “practical” tips that are often omitted in previous guides because researchers tend to learn them as they get more experience in the field and forget how useful they are for newcomers. Advice from this paper focuses on areas submitted to the workshop, like child-robot interaction, medical contexts, and human-robot teaming. Therefore, this paper will be especially useful to researchers who are newer to HRI, graduate students, early career researchers or those seeking to learn about one of the many aspects of the multidisciplinary HRI field with which they have limited experience. As Section 4 provides the practical tips, guidelines and specific examples extracted from the workshop, researchers can read the subsections of this section which are relevant to them, and adapt these tips and guidelines to their own research.

## 2 Materials and Methods

### 2.1 Participants

We recruited participants that would form two groups: mentors and mentees. When we refer to workshop participants, we refer to both mentors and mentees.

To recruit mentors, we identified top researchers with diverse skills in research design and research topics from diverse locations (United States (3), Europe (3), Asia (2)) with whom the workshop organizers had connections, Eight of nine mentors identified agreed to participate. Workshop mentors include Drs. Cindy Bethel, Hung Hsuan Huang, Selma Šabanović, Brian Scassellati, Megan Strait, Komatsu Takanori, Leila Takayama, and Ewart de Visser, with expertise in areas of real-world study, empirical lab study, questionnaire design, interview, participatory design, and statistics. We chose these mentors because of their valuable experiences, volunteered positions in the field each year, and their ability to advise new members in the field.

We invited potential mentees from across fields in HRI (e.g., computer science, ethics, robotics, psychology) who wanted feedback on their study design with quantitative or qualitative methods and statistical analysis. The advertisement included the names and expertise of the mentors. We advertised through direct contact with researchers and through email to HRI and HCI listserves, posts on social media, and early researcher forums, like previous year Pioneer Workshop participants (an HRI workshop for early-career researchers). We welcomed scholars at all stages of their careers, especially early career researchers to submit. We reviewed papers for relevance to the HRI field. We gave preference to papers that described their methods in enough detail to thoroughly critique and papers with methods that had not yet been conducted (except as pilot studies). In total, we accepted 16 papers to the workshop. See Table 1 for the keywords of the papers and their frequency. The geographical locations of the mentees were as follow: two mentees were from Kazakhstan, and the rest of the six mentees were from France, Germany, India, Netherlands, United Kingdom and United States.

**TABLE 1:** The keywords and their frequencies from the 16 submissions (^*^denotes keywords from authors who contributed to the paper).

Keyword	Frequency	Keyword	Frequency
Child-robot interaction^*^	5	Interdisciplinary^*^	1
Human-robot interaction^*^	4	Language learning^*^	1
Trust	3	Mental Model	1
Human-robot teaming	2	Multimodal explanation	1
Robot-Assisted Therapy^*^	2	Multimodal sensing^*^	1
Children with Autism^*^	2	Navigation	1
Acceptability^*^	1	Non-Expert User^*^	1
Adaptive instruction	1	Pain Management^*^	1
Anthropomorphism^*^	1	Parental inclusion^*^	1
Artificial Social Intelligence	1	Programming by Demonstrations^*^	1
Coaching	1	Reciprocal peer tutoring^*^	1
Collaborative and social computing devices^*^	1	Reinforcement Learning^*^	1
Computer systems organization^*^	1	Scene understanding	1
Emotion Recognition^*^	1	Social attributions	1
External interfaces for robotics^*^	1	Social robot^*^	1
Group Dynamics^*^	1	Socially assistive robotics	1
Healthcare^*^	1	Tactile perception	1
Human Factors	1	Team Innovation Capability^*^	1
Human-AI Teaming	1	Team Performance^*^	1
Human-centered computing^*^	1	Technology acceptance	1
Humanoid Home care	1	Theory of Mind	1
Intent prediction	1	Understandability	1
Interactive explanation	1	Wellbeing assessment^*^	1

### 2.2 Before the Workshop

We asked mentors to provide the keywords that describe their expertise. Approximately 2 weeks before the workshop, we divided workshop participants into groups of two mentees working with one or two mentors based on the mentees’ preferences and keywords in their paper that describe their works as well as mentors’ expertise. Groups received workshop papers from all group members and attended the workshop prepared to discuss them. Each mentee had a primary mentor, who read the paper in-depth, and a secondary mentor, who read the paper as time allowed. Because the workshop was online, we divided it into two sessions to accommodate attendees based on time zone, with the US and East Asia in one session (7 mentees, 8 mentors), and others from the US and Europe (9 mentees, 6 mentors) in another session (Table 2).

**TABLE 2:** Time table for Session 1 and Session 2.

Session 1 (United States and East Asia)			Session 2 (United States and Europe)		
MT 17:00	JST 09:00	Opening remarks	MT 07:00	GMT 14:00	Opening remarks
MT 17:15	JST 09:15	Breakout mentoring 1	MT 07:15	GMT 14:15	Breakout mentoring 2
MT 18:15	JST 10:15	Coffee break	MT 08:15	GMT 15:15	Coffee break
MT 18:30	JST 10:30	Individual work-time/ask mentor	MT 08:30	GMT 15:30	Individual work-time/ask mentor
MT 19:30	JST 11:30	Whole group discussion: lessons learned	MT 09:30	GMT 16:30	Whole group discussion: lessons learned
MT 20:00	JST 12:00	Closing remarks	MT 10:00	GMT 17:00	Closing remarks
MT 20:15	JST 12:15	Break until Session 2	MT 10:15	GMT 17:15	Workshop end

### 2.3 At the Workshop

We ran the workshop through Zoom videoconferencing. We started each session by introducing the workshop and how it was different from most workshops due to its in-depth critique of each accepted paper. Then, mentors introduced themselves and the main area(s) in which they would mentor during the workshop.

**FIGURE 1:**
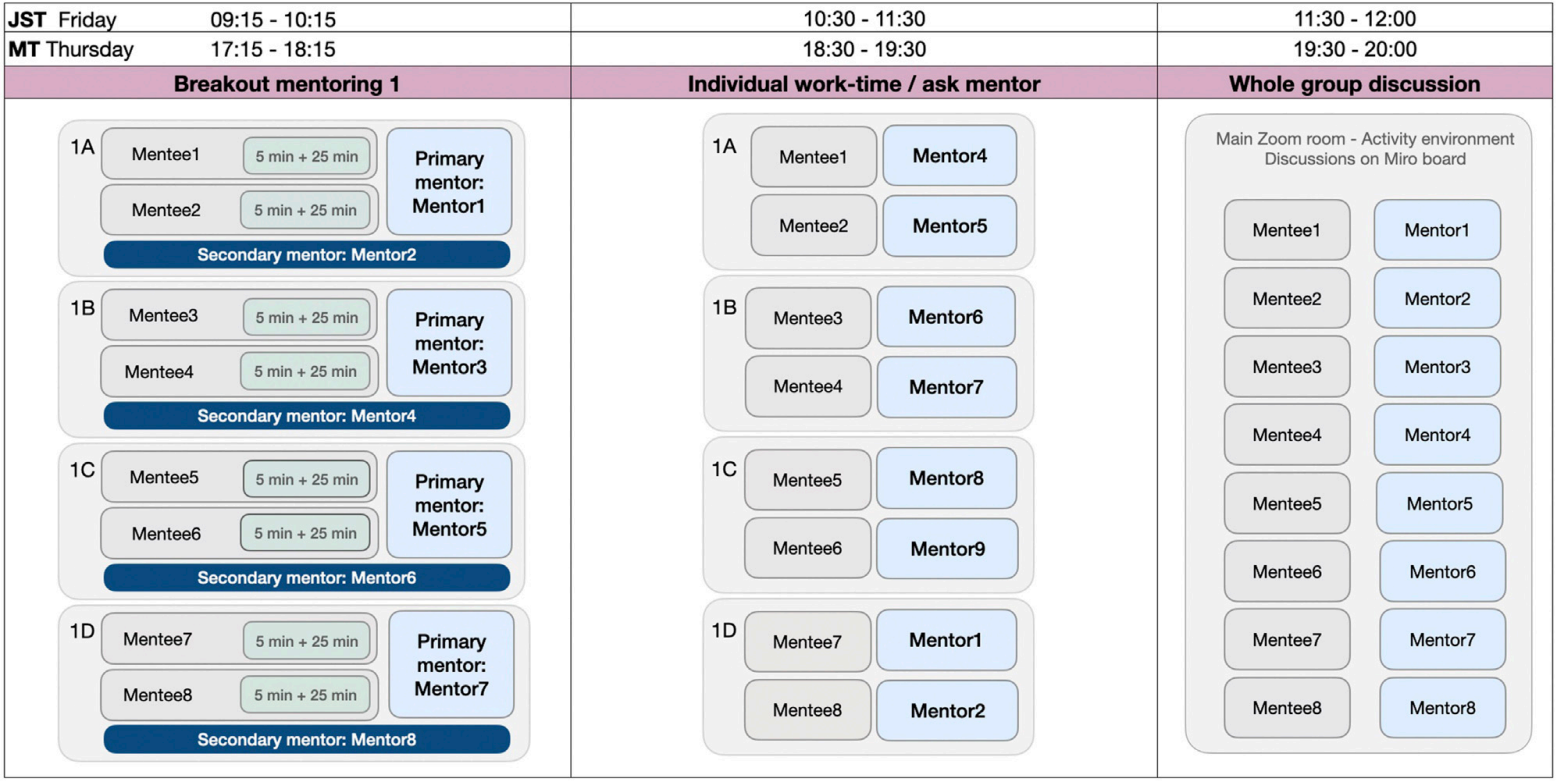
The workshop sessions included Breakout mentoring (main discussion with a main mentor, secondary mentor and two mentees), Individual work time/ask mentor (individual working, discussing and asking questions with different mentors) and Whole group discussion parts. A, B, C and D in Breakout mentoring and Individual work-time/ask mentor parts refer to the breakout rooms. Times are written in Japanese Standard Time (JST) and Mountain Time (MT).

The session broke out into smaller groups working with mentors. We created four breakout rooms including two participants, a main mentor, and a secondary mentor (Figure 1). In the Breakout mentoring session, primary mentors gave in-depth feedback and comments based on their thorough reading of the paper. Secondary mentors gave comments to provide another perspective and deepen discussions.

Each breakout mentoring session was a 60-min discussion session in which two papers each used 30 min to discuss and receive feedback. First, mentees gave a 5-min presentation to their group to summarize their work and the points that they wanted to get feedback on. Then mentees and mentors discussed the paper for 25 min. We instructed groups to focus on ways to improve the methodology, which could include, but was not limited to, study design of between or within participants, convincing control conditions, reducing confounding variables, appropriateness of measures and proposed statistics, improving scripts or questionnaires, and related research to read and cite.

Next, we ran the Individual work-time/ask mentor session (Figure 1). We grouped participants with different mentors with relevant expertise. In these 60-min session, mentees edited their papers and talked with mentors to continue conversations they began and asked questions as they arose. Mentees and mentors could move between breakout rooms to create the conversations that were most interesting to them. When many participants wanted more information about a specific topic, we created a room dedicated to that topic, which participants could enter and leave.

At the last part of the workshop, in the Whole group discussion sessions (Figure 1), participants gathered in the same virtual room and shared what they learned, including challenges and solutions to improve their methodology, by using a Miro^2^ board. In real-time, we worked with participants to cluster types of lessons together to create an overall summary of lessons learned from the workshop, which form the main contribution of this paper and we will discuss in Section 4.

### 2.4 Gathering Feedback

At the end of each session, we asked mentees and mentors to complete a questionnaire to provide feedback about the workshop and suggestions for future editions. Participants answered eight questions to assess key benefits we were interested in (e.g., would they keep in contact with mentors/mentees, did the mentees make substantive changes based on the workshop). Participants answered questions on a scale from 1 (“Strongly Disagree”) to 5 (“Strongly Agree”). Mentees and mentors also answered free response questions about what they found most valuable in the workshop, what they would like to stay the same, and what they would change if the workshop were run again. Out of 24 participants, 20 provided survey feedback.

**FIGURE 2:**
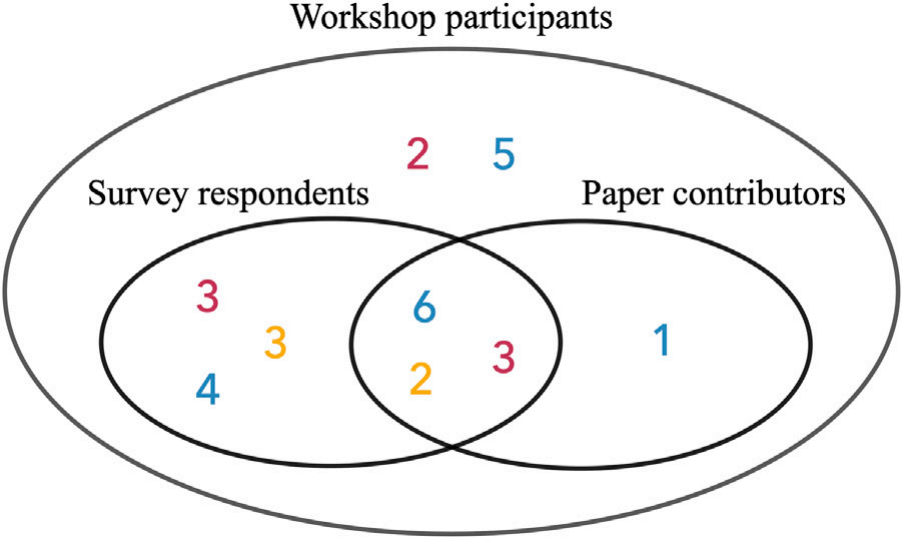
In total 29 participants participated to the workshop. 21 of them filled out the survey, and 12 of them contributed to this paper (red: mentor, blue: mentee, yellow: mentee’s colleague).

We also invited participants to contribute to this paper by writing about the lessons they learned from the workshop. Interested participants used themes based on clusters in the Miro board and drew from their discussions during the workshop to contribute to the lessons learned section in the results below. Then, mentors read the lessons learned to add to and clarify points the mentees wrote about (Figure 2).

### 2.5 After the Workshop

The organizers created potential lessons learned topics from the overall summaries that were created at the Whole group discussion sessions. In total, the organizers put 25 topics on a Google Form. Then, participants (mostly mentees) completed a survey indicating who wanted to contribute to this paper and which topics they wanted to write about. The workshop organizers assigned participants to one to two sections such that participants got their first, second, and/or third choice. Then, participants took approximately 1 month to write their sections. During this 1 month, the organizers conducted three online meetings to discuss the mentees’ questions about their parts to be included in this paper. Finally, the workshop organizers edited the sections for flow and content and invited mentors to review the paper to ensure that the paper accurately reflected the lessons learned.

## 3 Results and Discussion: What People Liked and How to Improve the Workshop

In this combined results and discussion section, we present participants’ feedback about the workshop. We collected participant perceptions of the workshop through a survey, which included Likert scale responses and free response. We report both of these below. Overall, participants found the workshop valuable, especially their interaction with multiple mentors. To improve the workshop for future years, they suggested including more contact with other participants, such as through small breakout rooms on common topics (e.g., advice on what can go wrong in real-world experiments of vulnerable populations). We discuss these in-depth below.

### 3.1 Likert Scale Responses

**FIGURE 3:**
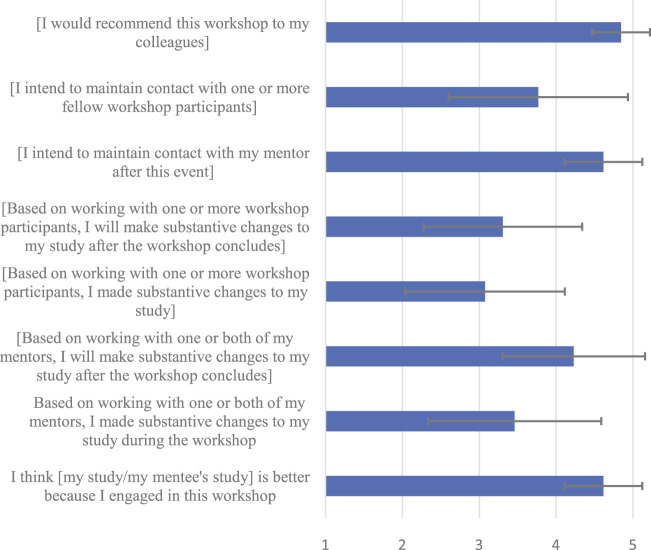
Survey results means reported. Error bars represent standard error. Participants reported answers on a scale from 1 (Strongly Disagree) to 5 (Strongly Agree).

We surveyed workshop attendees about their attitudes toward the workshop (Figure 3). Because mentors (*N* = 6) and mentees (*N* = 14) gave similar responses, we combined their answers when possible to report below. Mentors and mentees would recommend this workshop to their colleagues and thought that the studies improved because of the workshop. They indicated that based on the mentor-mentee relationship, substantive changes to the study occurred during the workshop, but were even more likely to occur after. Mentee-mentee interaction did not particularly improve the papers. Mentees intended to maintain contact with their mentors after the workshop and vice versa.

### 3.2 Free Response About Best Benefits and Things to Improve

We asked attendees free response questions regarding what they liked about the workshop and what they would improve. One author read through the free response questions to determine themes using a bottom-up approach. Then, that author categorized the responses according to the themes. The three themes that emerged were: 1) Attendees liked and wanted more break-out group time for interaction between mentees and mentors, 2) They suggested having more interaction between workshop participants, especially on common themes in their studies, and 3) They recommended having small breakout rooms about specific topics to facilitate discussion on common issues, such as study design, power analysis, and managing expectations. We discuss these further below.

#### 3.2.1 Small Group Discussion With Mentors

Attendees indicated that time and advice from mentors were the most valuable part of the workshop (12/20). They indicated that in future iterations of similar workshops, they would like to keep the small group time with mentors (11/20; “The personalized feedback from the mentors was really useful!” Anouk Neerincx^3^). Attendees indicated that they liked having multiple mentors (6/16), and several participants indicated that they would like more mentors (3/20; “I’d like more mentors to take a look at my work, perhaps through a rotation.” Michelle Zhao). Attendees liked the amount of time spent with mentors (4/20), and some wanted even more time with mentees and mentors (3/20; “… more time on the live one-on-one interaction” Ewart de Visser).

#### 3.2.2 Time and Discussion With Mentees

Some attendees recommended including more time to connect with other participants about their work (4/20; “A time period for mentees to ask questions and give suggestions about the other mentees’ papers.” Anonymous. “As an extension of the lessons learned, one could try to link-up participants that face similar challenges and run a short (15min) breakout session to encourage further contact.” Anonymous).

#### 3.2.3 Specific Topic Discussions

Several attendees indicated topics that were most helpful to them: study design (4/20) and issues unique to their study (4/20; e.g., “Advice on things that could possibly go wrong in real-world experiments when working with vulnerable populations and managing expectations.” Sudhir Shenoy). A couple of attendees indicated that they would enjoy having breakout rooms about common topics (2/20; “Maybe also having a specific “class” portion. For example, we had a spontaneous lesson on how to conduct a power analysis, which was very helpful.” Anonymous.).

## 4 Lessons Learned About Designing Studies for an Human Robot Interaction Audience

**FIGURE 4:**
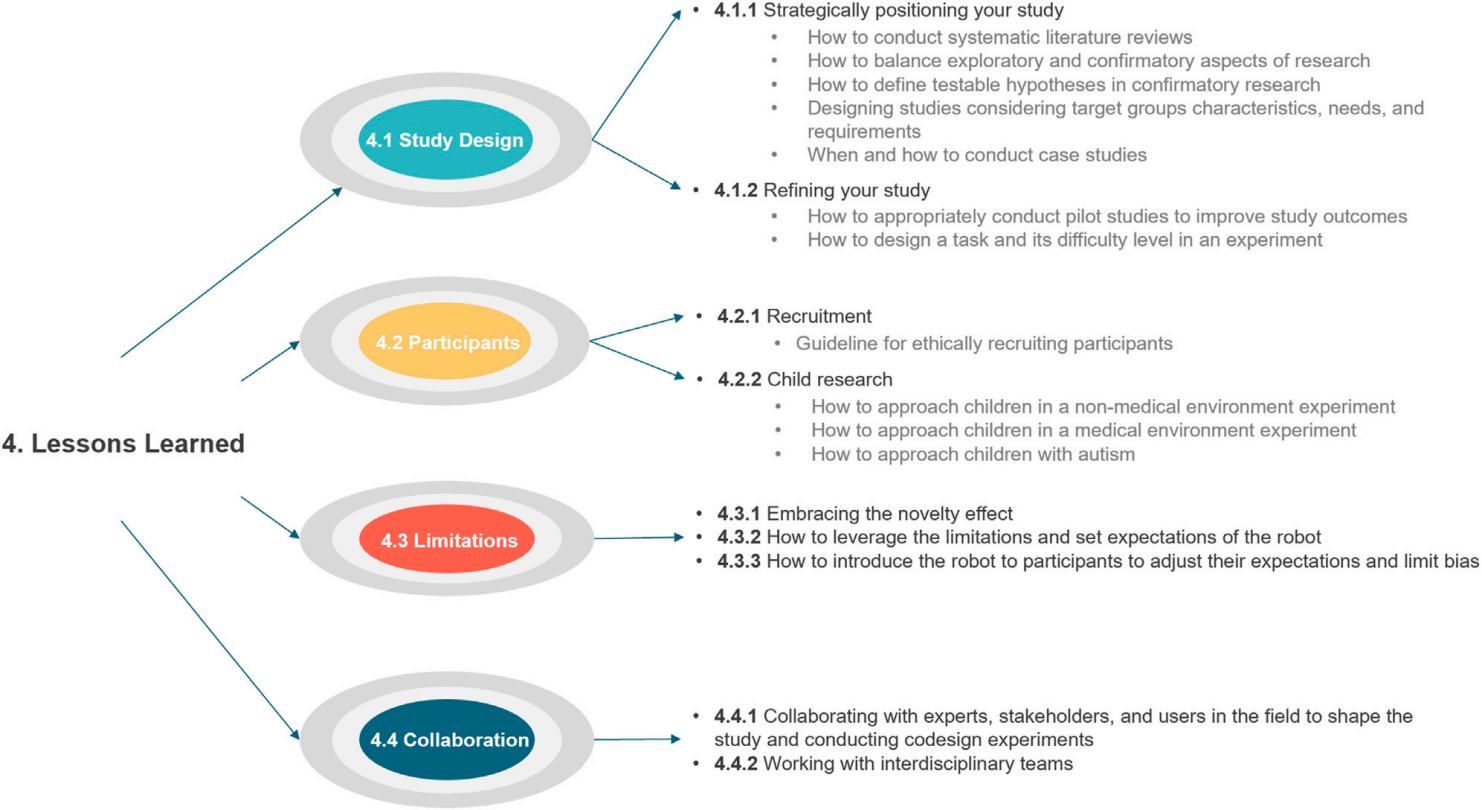
Graphical representation of Lessons Learned topics.

The end of the workshop included whole group discussions of lessons learned. The workshop organizers extracted topics from these discussions of the studies from the child-robot interaction, robot-assisted therapy, human-robot teaming, and children with autism because these were the most common themes in the workshop papers (Table 1). We use these topics to develop four themes discussed below: study design, participants, limitations, and collaboration. Figure 4 shows the graphical visualization of these topics and related subtopics. We discuss these in-depth below.

The workshop participants who are also authors wrote these sections based on what they learned. The workshop organizers and mentors assisted editing the sections for clarity and flow. While some sections below refer to citations, other sections have few if any. This is because the information here comes from workshop participants and what they learned from the mentors. Much of this information is based on first-hand experience that has not been published. Thus we include this section in the results section: because some of this information is new (unpublished) information that arose from the discussions during the workshop. We hope that in the future, scholars will be able to refer to this work when deciding on or justifying certain study methods.

### 4.1 Study Design

When designing a study, there are many possibilities and many places to start. In this section, we discuss how researchers can strategically position their studies to be the most beneficial to the field and application through systematic literature review, balancing exploratory and confirmatory aspects of research, and defining testable hypotheses for confirmatory research. We then indicate how researchers can refine their study through pilot tests, especially of task difficulty.

#### 4.1.1 Strategically Positioning Your Study

Before even deciding what to study, researchers need to think about how to strategically position their studies to be of most use to the research community and people who will actually use the technology (also see Collaboration section). Researchers can begin to narrow in on specific research ideas by learning more about what others have already studied in the field through systematic literature review. Such review will also help researchers understand if their area of interest is open or novel enough that an exploratory study could uncover new important themes, or if they can conduct studies to support existing theories. Below we discuss how to conduct systematic literature review, balance exploratory and confirmatory aspects of research, define testable hypotheses and confirmatory research.

##### 4.1.1.1 How to Conduct Systematic Literature Reviews

Researchers use systematic literature review to develop a well-defined research question by critically analyzing seminal works, influential theories, and fundamental questions in a particular knowledge domain. Through systematic literature review, researchers learn what others have already studied in the field. In doing so, they can identify specific research ideas, promising themes, replicable methodologies, and major research gaps, as starting points for exploration or to direct how they test their current research questions. For researchers who wish to publish a systematic literature review, we also recommend that they learn the Preferred Reporting Items for Systematic Reviews and Meta-Analyses (PRISMA) method, which is meant to help authors improve reporting of literature reviews (Moher et al., 2009; Moher et al., 2010).

A good way for researchers to start a literature review in a new field can be with books, textbooks, review articles, and meta-analyses that overview the field. Then, researchers can check the reference list and find additional relevant research works, or use “cited by” features in search engines to find more recent articles that cited these seminal articles. It is good practice to read numerous articles in the area until scholars get a good sense of what common themes and seminal articles the researchers typically discuss. In cases when they cannot access specific articles or necessary measures, they might contact the researchers to request a copy of the article or measure - but some researchers may not be able to provide this, especially if the research is more than five or 10 years old. Sources like these can help develop a more complete understanding of what research has already been done and may reveal open questions.

As researchers narrow into their specific field of interest, they can take inspiration from previous studies in HRI and related fields. In areas that are more developed in HRI, researchers could replicate and extend work from a recent paper. In areas that are newer to HRI, researchers have to draw more from related fields. For example, researchers exploring HRI applied to building innovation capabilities within organizational settings, might start from “team-level studies” in human-human interaction because innovation is increasingly emerging as a team-level phenomenon within humans’ organizational settings (van Knippenberg, 2017). Team innovation examines the ability of (human) teams to transform their inherent knowledge, skills, and abilities into innovative products or services (Drach-Zahavy and Somech, 2001). Inspired by these sources, researchers might investigate the effects of including robots in such innovation teams on team processes like team interaction, task efficiency and, effectiveness. Such endeavors can help researchers find potential answers to some of the fundamental questions in their field and run studies that provide insight into human-robot and human-human interaction, such as 1) Can robots spark creativity in (human) teams? 2) Can robots help (human) teams to improve their ability to innovate within organizational settings? 3) Can teaming up with robots improve the ability of (human) teams to ideate, effectively, and efficiently?

A systematic review of literature is the foundation for developing and answering strategic questions that advance the current understanding through novel ideation, contextualization, or experimentation. As researchers learn more about the type of work in a particular subdomain, they can also determine how much of their research should be exploratory versus confirmatory to most effectively advance the field.

##### 4.1.1.2 How to Balance Exploratory and Confirmatory Aspects of Research

Exploratory and confirmatory research can both contribute to scientific progress. Typically, studies in a subfield progress from exploratory research in a novel area to confirmatory research to support findings from exploratory studies. However, exploratory studies can also bring novel perspectives to a well-studied area. We discuss the two types of research below.

Exploratory research. In exploratory research, scholars examine relationships underlying observations or phenomena, *without a priori hypotheses*–that is, without hypotheses based on theory. Because exploratory research is very qualitative and uses a smaller sample size than confirmatory research, scholars should be especially cognizant of their study design to improve confidence in interpreting outcomes. Researchers can do this through predetermined testing criteria and adequate sample sizes. Exploratory HRI research can expand the scope of the domain by incorporating and integrating with emerging domains, dimensions, and disciplines of inquiry. For example, researchers attempting to conceptualize a novel construct such as Human-Robot Team Innovation Capability (HRTIC) and measure it through a psychometric scale, must first conduct an exploratory study to identify the potential sub-dimensions of HRTIC. Such exploratory studies can introduce fresh perspectives, unique variables, and novel experimental designs to develop theories in the ever-expanding field of HRI.

Confirmatory research. In confirmatory research, scholars test a particular theory with *a priori hypotheses*, to learn more about cause and effect. Confirmatory research can help the HRI field through rigorous theory-testing. Researchers often run a balance of studies under strict conditions to promote the study’s validity, internal reliability, and replicability, and studies under more diverse conditions to examine external ability and generalizability. Through these studies and statistical testing, researchers can find support or lack thereof for the hypotheses (but see articles on the dangers of statistical testing; Greenland, 2019; Trafimow, 2019; Zhu, 2012). To do so, researchers need to ensure they have an adequate sample size, a predetermined power of at least 0.80 (see Brysbaert and Stevens, 2018 for more details), and report effect sizes. For example, if researchers had already created a psychometric scale based on exploratory studies of HRTIC (described above), they should validate the scale through confirmatory analysis. This might involve testing the scale on several different populations of interest and measuring if it predicts other measures of HRTIC or other outcomes related to HRTIC.

##### 4.1.1.3 How to Define Testable Hypotheses in Confirmatory Research

In confirmatory research, experimenters must define *a priori hypotheses*, or what patterns they expect in the results based on a theory or previous research. This is critical because it defines the scientific purpose of the study, or what the researchers seek to confirm. Defining clear, testable, and an appropriate number of hypotheses provides a strong foundation to interpret study results.

The research question directs the experimenters in defining their hypotheses, independent variables (if any), tasks, and the measures to support or reject the hypotheses. Hypotheses should be specific enough to enable their verification (or rejection) so researchers can draw relevant and meaningful conclusions. Defining the independent variables and measures after identifying the research question may not be trivial depending on the chosen hypotheses. One good way to define them is to follow three phases: 1) Think about the research question(s) behind the hypotheses (e.g., “Does being in a group, rather than alone, influence perceptions of robots?”), 2) Choose appropriate independent variables and measures corresponding to the ideas (e.g., groups will be three people, alone will be one person. We will specifically measure trust of robots using the Multidimensional Measure of Trust; MDMT (Ullman and Malle, 2018), and 3) Write the hypotheses to verify by the chosen measures (e.g., “Participants’ trust of a robot alone will differ from their trust of the robot after interacting with a group”). Once the hypotheses are defined, the experimenters can prepare the corresponding task. This process is also iterative and circular, and researchers may go through various steps multiple times as they design and hone the research study. Researchers can see (Chernova and Thomaz, 2014) to learn more about defining hypotheses.

The number of hypotheses should be small such as under four. Too many hypotheses lead to huge experiments because hypotheses define the number of experimental factors, which define the number of participants and increase the duration of the experiment. Many hypotheses may create an unclear study. Researchers who wish to answer many research questions may benefit from conducting several smaller experiments to assess a few hypotheses at a time.

Experimenters must define their hypotheses before conducting the experiment and not modify them after getting the experimental results. This is important; modifying hypotheses after obtaining the experimental results creates a false impression that the study confirmed an *a priori hypothesis* and can mislead researchers in the field. Even if all hypotheses are rejected, it is a good scientific contribution that may help other researchers by showing when a particular theory fails or that a specific manipulation is not strong enough to produce the expected results. One good way to do this is through pre-registering one’s hypotheses through initiatives such as https://www.cos.io/initiatives/prereg. Websites like these allow researchers to indicate their hypotheses in advance of running the study so that when they publish the results later, other scholars are confident that they did not change the hypotheses after observing the data.

##### 4.1.1.4 Designing Studies Considering Target Groups Characteristics, Needs, and Requirements

It is critical to account for the needs of their participants based on their demographic groups and the context of the study. Researchers should consider this closely while designing studies to ensure that their participants can fully participate and can provide useful data, such as by avoiding fatigue effects or failure to respond due to discomfort.

Researchers should attend to the target group’s characteristics, needs, and requirements that vary across different demographic groups (Sandygulova and O’Hare, 2018). Each target group has unique challenges, and researchers must be aware of them before designing user studies. For example, in designing studies with children, maintaining task engagement throughout the experiment is more important than in studies with adults. Researchers might consider incorporating many breaks into their study design with children to maintain task attention throughout the experiment session (Masini et al., 2020).

The target group’s characteristics can also affect how researchers define their variables or their target population. For example, children have developing cognitive systems, which researchers need to account for in determining what age range of children to recruit. An age range of 2 years might be considered too broad while designing experimental tasks for children. However, an age range of 5 years might not be considered too broad while designing studies with adults. Even within the same age groups, there is a difference in cognitive abilities between girls and boys. Thus, researchers must account for the developing cognitive systems to avoid inconsistencies in research findings and interpretations.

During the experiment, it is important to create a safe, collaborative environment, especially when working with vulnerable populations (e.g., children, adults in cognitive decline). Coming up with ground rules together with the participants at the start of the experiment could help. This may be as simple as having drinks and food present. It may also be a good time to define topics that participants are not willing to talk about, to avoid discomfort.

Researchers also face challenges depending on the context in which they will run their study. For example, in designing healthcare studies, the experiment design needs to align with existing therapies and treatments. Researchers can ensure this by speaking with experts (clinicians, therapists, and physicians) to determine what target goals would be appropriate (e.g., function level, age) and develop studies informed by the existing and accepted methodologies (see Collaboration section for details).

##### 4.1.1.5 When and How to Conduct Case Studies

There are many types of studies that researchers can run to gather exploratory and confirmatory information, including observational studies, focus groups, interviews, experiments, and more. Scholars interested in these can find details on running such studies in psychology and human-robot interaction textbooks (e.g., (Bartneck et al., 2020; Shaughnessy et al., 2000). In this paper, we draw attention to case studies, which can be a particular challenge, especially in human-robot interaction.

The case study is a predominantly qualitative research approach in which researchers study one situation or one participant in great detail. It enables researchers to gain a deeper understanding of complex phenomena in real-life contexts. Researchers from social sciences use it more often compared to their counterparts in other fields. It involves a wide range of empirical data collection tools like observation, interviews, focus groups, and personal narratives as documentary evidence. It has been criticized for its subjective nature and lack of scientific rigor, having a less solid basis for generalization of findings to larger groups and settings (Crowe et al., 2011), but it can also be valuable for unique situations from which researchers could not collect data from more cases (e.g., a very rare illness). Researchers should consider waiting until later stages of their academic HRI careers before conducting case studies because:

New researchers in the area will have more trouble breaking into the field with a case study than with the more accepted empirical study that has a sizable sample. This is because of the history of considering case studies as an illegitimate, less rigorous research strategy, and because there are few case studies in user-centered HRI. Researchers who do choose to use case studies must have strong justification for doing so. For example, researchers may opt for case studies when their research interest is under-researched, when the technology studied is novel and inaccessible (Endsley et al., 2017), or when they work with hard-to-retain and vulnerable populations (e.g., individuals with autism or learning disabilities), and when qualitative data is necessary.

Further, case studies usually take a longer time to observe individuals at great depth in their natural environment, which can delay publications and be detrimental to early-career researchers. The collected data may be overwhelmingly large and multi-faceted. Data collected in the field consists of different settings including personal spaces like homes. Every individual or target group needs considerable attention at each stage of research. Observation is key to evaluating behaviors in-depth both during controlled experiments and naturalistic interaction but can take especially long to code and prepare for analysis. Researchers who choose to conduct case studies should identify research phases in advance according to systematic protocol and a clear vision of what kind of questions to explore. They must also conduct ongoing reporting and documentation of their observation in an organized and reflective way.

Finally, case studies will have additional challenges compared to the typical empirical study because as time passes, situations change, and researchers’ data-collection methods may need to change. Researchers should be flexible and remain open-minded to accept that they will not always make the best decisions along the way because the method is primarily based on subjective judgments and human perception of past events.

#### 4.1.2 Refining Your Study

After strategically positioning one’s study through systematic literature review, balancing exploratory and confirmatory aspects, defining hypotheses, and designing the study, it is critical to refine the study. Researchers often do this through pilot testing–that is, testing out their procedures on participants, especially with similar attributes to the population. We discuss this below in general and in relation to designing a task and its difficulty level.

##### 4.1.2.1 How to Appropriately Conduct Pilot Studies to Improve Study Outcomes

Researchers can perform pilots at all levels, from testing out a portion of their studies on the experimenters themselves to testing the entire study on people from their population. Scholars should usually perform multiple pilot tests at different levels as they refine their studies. How they do so in each instance likely depends on which aspect of the study they want to address: issues with the technical robot system and room, unexpected participant behavior, and issues with the study design, process, or metrics, and more.

For technical reasons, researchers should evaluate the robotic system not only in the lab but where the main study will take place (e.g., a hospital or a shopping mall). Outside of the lab, robots can behave unexpectedly for general reasons, such as changes in lighting or noise level - which can affect the robots’ programming - and sturdiness or angle of the floor - which can affect a robot’s ability to move appropriately. They can also behave unexpectedly for study-specific reasons, such as older adults not hearing or understanding the robots’ voices. Pilot testing can also reveal any safety-related issues (e.g., lack of fresh air in the experimental room, placement near a fire hazard).

Testing robotic system functionality in a pilot study with a few target participants (e.g., children with autism) can reveal unexpected participant behavior. For example, children may press a NAO robot’s chest button which makes NAO utter a loud message about its IP address. Knowing about such behaviors in advance of the main study allows researchers to address them early, for instance by disabling such messages.

Additionally, a pilot study can reveal problems with the study design, process, or metrics. Recruiting subjects or collecting data could be impractical or too expensive or time-consuming to manage. Testing a questionnaire can reveal if participants understand the questions and check if researchers are measuring what they intend to measure. Running trial data analysis on pilot or simulated data can also help to understand if the proposed analysis is appropriate for the data.

Pilot studies can be so valuable that sometimes the research community can benefit from researchers publishing their pilot studies with a focus on study feasibility, the approach taken, and lessons learned to save money, time, and resources of other researchers.

##### 4.1.2.2 How to Design a Task and Its Difficulty Level in an Experiment

One part of the study that is especially important to pilot is the task. The task defines what the participants will do during the experiment. It needs to allow researchers to appropriately assess their hypotheses, be highly feasible for the participants and robots, and researchers need to document it for future scholars.

A good way to choose an appropriate task that allows researchers to assess their hypotheses as feasible is to select one that has been tested and validated in the existing relevant literature. Still, researchers should pilot test the task in their specific lab with their specific participants. This is because experimenters must prepare for subtle but important differences between their study, participants, or experimenters than the original study. If researchers cannot select a previously-validated task, they may create their own. In this case, researchers should engage in more in-depth pilot testing by conducting multiple tests of the entire task (see Section 4.1.2.1 on how to appropriately conduct pilot testing). Researchers can also include manipulation checks to assess if participants perceived the task to be of the intended difficulty level that the experimenters intended. They may do this by including a question at the end of the study or after the task to assess task difficulty or anything else they want to know about the task.

Optimal task difficulty depends on the purpose of the study, the participants, and the robots. The experiment should be feasible for participants and not create a large workload or physical difficulty (unless researchers are specifically studying difficulty). Researchers can directly ask participants how difficult the task was for them, but participants may not wish to admit if they found the task difficult. Researchers can get more accurate answers if they emphasize to participants that they are testing the research task, and if the task was difficult for any given participant, it will likely be difficult for the others. When researchers cannot simplify a difficult task for participants, they may need to train all the participants. A training phase can reduce the impact of participants’ differential incoming skill levels on the results.

The task should also be feasible for the robots, which have different functionalities and abilities based on their design. Robots can have limitations in their load capacity, communication skills, sensors, motors, and more. If the task is too difficult with the robot, researchers should simplify it or choose another task.

Finally, researchers should carefully document the task and the entire experiment so other researchers can replicate the study with the same conditions and a similar environment. Within the HRI community and the scientific community at large, there has been a stronger emphasis on the ability to replicate studies to build generalizable knowledge. Some in the HRI community have for example noted difficulties in replicating established effects in HRI such as social facilitation (Irfan et al., 2018), social desirability (Leichtmann and Nitsch, 2020), and trust (Ullman et al., 2021). This concern extends across the field of psychology and relates to ongoing debate about the “replication crisis” (Amrhein et al., 2019; Baker, 2016).

### 4.2 Participants

One critical component of any HRI study is the human participants. Below, we describe and provide advice about identifying and recruiting participants, and about working with participants in special cases: medical settings and child research.

#### 4.2.1 Recruitment

##### 4.2.1.1 Guideline for Ethically Recruiting Participants

Participant recruitment is multifaceted, and researchers must do so ethically. They should obtain ethical approval for recruitment and permission from managers of recruitment locations. Researchers can recruit more effectively by considering the study location. They should also make sure participants are not overly induced to participate in the study by ensuring that they frame the study as voluntary and provide accurate information about the study. We describe these in-depth below. We point out that specific guidelines may vary from country to country and institution to institution. We include the section in the paper is one example of good guidelines, but we encourage authors to seek out specific guidance from their own institution and ethics board.

The participant recruitment process begins with obtaining ethical approval for the study and recruitment procedure. Typically, researchers seek approval from an Institutional Review Board (IRB), which assesses the researchers’ plans to ensure that they follow ethical guidelines. Researchers describe their proposed plan for recruitment and provide recruitment materials for the ethical board review. Such materials typically include recruitment emails, phone scripts, social media postings (e.g., Facebook or Instagram that could be via either personal account, university or school’s accounts), printed posters placed around recruitment grounds, and others. A typical text for recruitment should indicate the researchers’ names and contact information, the purpose of the study, eligibility and/or ineligibility criteria, (briefly) what potential participant would do (e.g., a 30-min interaction with a robot), and incentives if any (e.g., monetary payment, a cup of coffee, etc.). Ethical committees often provide templates for such recruitment materials. Researchers should be mindful of how they advertise the study because participants can self-select, which may create a biased sample (Foroughi et al., 2016). For example, if researchers indicate that the study is about robots, they may over recruit from people with strong opinions about robots (positive or negative).

Researchers often need to seek permission from those managing the sites from which they wish to recruit. For example, researchers recruiting through a mailing list or social media platform should contact the administrator or marketing manager. Likelihood of approval may depend on who the media reaches - for example, a university’s student list might be more likely to approve recruitment in comparison to a faculty members’ list. Researchers recruiting K-12 children through the educational system must seek permission from the school principal, then from individual teachers to recruit via a specific class at a convenient time. Researchers recruiting from and running studies at other institutions (e.g., nursing homes, long-term care settings, hospitals, rehabilitation centers) must obtain permission from the institution’s administration or director. It can be helpful for researchers to have a conversation with the manager of any site they wish to recruit from to understand how they can respectfully recruit participants. Often, the ethical committee requires written consent from such managers or directors to recruit from such media or external settings before approving recruitment.

A study’s location affects how research can effectively recruit participants. In non-campus laboratory studies, researchers could recruit nearby participants via posters in an elevator or at a building entrance so participants would not spend much time commuting to the experimental site. Conversely, for a study that is far from campus, it may not be effective to recruit participants from a residential campus who may not have access to cars or transport. For experiments conducted in public places, like museums, train stations, or shopping malls, researchers could recruit by approaching people in a standardized way (e.g., every tenth person through the door) and using an oral script. In these cases, researchers might ask the ethics board to allow participants to give oral, rather than signed, informed consent to speed recruitment and enhance the chances that people have time to participate.

During recruitment, researchers must show that participation is voluntary. For example, faculty members or teaching assistants should avoid recruiting students from the classes they directly teach because students may feel obligated to participate. In addition to violating the voluntary nature of recruitment, this perceived obligation could create power relationships in the experiment. To reduce these concerns, a research assistant could recruit from the class without the presence of an instructor and store data such that the instructor cannot find out who participated. Similarly, if a researcher has a relationship (personal or professional) with a potential participant, the researcher must emphasize the voluntary nature of participation and that a decision to participate will not impact their relationship.

Researchers should help participants understand what the study entails including the risks and benefits. They can begin to do so by providing accurate information in the recruitment materials. This is especially important for research in healthcare settings because patients tend to believe that anything healthcare providers suggest could benefit them. Researchers should counteract such misconceptions by providing accurate information, including about potential risks, uncertainties, and threats associated with the experiments. They should remind participants that they can drop out of the experiment at any time. In addition to providing this information, researchers must give participants enough time to consider the decision. Some research must include deceiving participants about the true purpose of the study. For example, knowing that the robot will ask participants increasingly ridiculous requests until the participant refuses to complete the request would have a dramatic effect on participant compliance. In these cases, researchers can still provide as accurate information about the risks and benefits as possible before the study and debrief participants after the study with information they could not provide before the study.

These are several ways that researchers can be aware of possible ethical issues in recruiting participants. Researchers should also consider other ethical concerns in recruitment, such as equitable selection of participants and respect for privacy with and vulnerable circumstances (e.g., with the autism population).

#### 4.2.2 Child Research

HRI has historically included children and young users in studies with robots (Baxter et al., 2011; Charisi et al., 2016; Belpaeme et al., 2018; van Straten et al., 2020). Running studies on children has its own set of challenges. In the section, we address how to approach children in non-medical environments, medical environments, and how to approach children with autism.

##### 4.2.2.1 How to Approach Children in a Non-medical Environment Experiment

There are several things researchers should be aware of before they run experiments with children. The informed consent process is more involved, and the researchers need to account for differences in children’s cognitive systems, discomfort, unpredictable behavior, and attention span. We discuss these below.

Before beginning a study, the children need to assent to the study, and a guardian of the child needs to consent. Researchers can obtain this by sending written child assent and parental consent forms home with children and asking their teachers to collect them later that day or week. Children and their guardians should have a chance to ask questions about the experiment or robot. After the experiment, the researcher could debrief the children in a class format to explain how the robot works, provide a demonstration and allow them to ask further questions. The experimenter could debrief the guardians through a written document that includes the experimenter’s contact information in case the guardians have any questions.

Designing studies for children can be very challenging because researchers need to attend to children’s developing cognitive systems. Thus, all aspects of the experiment design (recruitment, protocol, and data acquisition) must account for differences across different developmental epochs (Sandygulova and O’Hare, 2018). Age is only an indicator for cognitive development, so researchers should (if possible) design tasks that are suitable/engaging/etc to a wide age group.

Researchers should take special care to avoid children’s discomfort. This is important because children may not have as much ability to cope with discomfort, or to disclose that they are uncomfortable, as adults. Further, parts of the protocol that do not seem unusual to experimenters can sometimes be an issue. For example, young kids may be upset being alone in a room if they have never been alone in a strange place before. If children are uncomfortable, the experimenter may have to terminate the study early. Researchers should make the experiment setup (room and protocol) comfortable for participants. Researchers can improve comfort by using data modalities that are less intrusive (e.g., play sessions and short surveys rather than physiological monitoring) and having an experimenter, or even the children’s guardians, in the room during the study to make the unfamiliar experimental setting less intimidating for children. When determining who might be in the room with the children, the researchers should consider how the presence of the experimenter or guardians might bias the children’s responses. To decrease children’s worry about giving incorrect answers, researchers should emphasize that they will not be assessed or graded on what they did or said and that there are no right or wrong answers. For example, the researcher could ask children their favorite colors and, as they answer, the researcher could stress that there is no wrong opinion. Appropriate briefing and debriefing must be planned for contingencies; if a child withdraws from the study, the child shouldn’t feel like it has done something wrong. Researchers should also talk to the children’s parents to learn about the children’s idiosyncrasies and improve comfort (e.g., avoid touching a child who is touch-averse), especially when working with children from special populations (e.g., on the Autism spectrum).

Another challenge in child studies is the unpredictability of children’s responses. Undertaking pilot studies with friends’ or colleagues’ children before the actual study helps researchers understand children’s needs and requirements in non-medical environments. Familiarisation activities like an introductory dance or story narration before the actual experiment may also help avoid the novelty effect with children. Researchers can also incorporate a semi-structured experimental protocol to account for the varied responses across different age groups and stages of the experiment setup. HRI researchers can use a structured Wizard of Oz (WoZ) setup, in which a researcher covertly controls the robot, to increase flexibility of robot behavior and maintain some spontaneity in responses. Researchers can also have children perform the experiment in groups of two and explain their reasoning to each other to gain deeper insight into the mindset of the children while they interact with a robotic agent.

Researchers should account for the limited attention span of the children and to maintain concentration on the task throughout the experimental session (Yamada and Kobayashi, 2018). Researchers can incorporate several breaks in their study design to make the study session more engaging and productive.

##### 4.2.2.2 How to Approach Children in a Medical Environment Experiment

Any HRI experiment in a medical environment is complex. They involve high-stakes of interacting with medical professionals and patients’ health. They also tend to be resource-intensive to create systematic procedures and protocols. Adding children to this complex design is even more challenging. Some main challenges of performing experiments with children in medical environments are the increased discomfort due to the medical setting and unreliability in participation.

Hospitalization can be unavoidably traumatic as children often undergo painful procedures in an unfamiliar environment. Child patients in the hospital long-term are often subject to a strict dietary regimen, monitoring, and scheduled procedures. This makes it even more important that researchers attend to the children’s comfort during the study. Researchers can increase children’s comfort and decrease their fear or anxiety by running the study in a neutral space, away from the procedure room or areas the children associate with trauma. The appearance of the room needs to be different from the procedure room to make the child feel comfortable. Researchers can differentiate themselves from the medical staff by wearing casual clothes different from staff uniforms. However, the parents may be less happy about research staff not wearing the uniform because they associate the uniform with competency.

Recruitment is often unreliable because children’s availability varies on many dynamic factors, like availability of medical equipment and personnel. Children may also be discharged sooner than predicted. Researchers should talk to all the caregivers of the child such as therapists, psychologists, parents, not just the doctors or nurses, to learn if they must exclude the children from the study for reasons other than the primary medical condition. This unreliability in participant recruitment and retention can cause delays in the study.

##### 4.2.2.3 How to Approach Children With Autism

In HRI contexts, researchers often ask children with autism to engage with a social robot to practice social, cognitive, and behavioral skills. We recommend using a couple of ways of approaching children with autism when conducting human-robot interaction research (Kim et al., 2012).

It is especially important to help the children with autism be comfortable so they can engage with the experiment. A researcher intending to work with autistic children should tailor the interactions to each child’s needs. They should learn how each child reacts to a new person and of behavioral patterns with which each child is familiar. To do so, researchers should volunteer to get to know idiosyncrasies and acknowledge the roles of stakeholders involved by talking to parents, therapists, teachers, and caregivers. For instance, a researcher may introduce themselves before the experiment and observe a child’s behavior in a regular setting.

Researchers should also include their target population in all stages of the research, including in interpreting results, to make sure they are most accurately interpreting the data. Some examples of participatory design approaches with autistic children have been reported recently (Millen et al., 2011).

Parental involvement is a great way to support children with autism during experiments so that they feel at ease and comfortable. Apart from parental self-reported data, their physical presence and occasional prompts may help children to get used to a new environment. Notably, parental presence may not have a good effect on all children equally; thus, a researcher could invite parents to initial experiments and observe if they can benefit both children and the study.

It is also good to know how children react to the robot before experiments. It would be beneficial to have a play-based experience, especially for preschool and primary-school children. The playtime should be kept as short as possible because children usually have a short attention span and may lose interest quickly. Playing with one favorite toy and constructing LEGOs are often a preferred pastime for children with autism. Also see the “How to introduce the robot” section.

### 4.3 Making the Most of Limitations

Because the technical advancements are not matured enough for robots to perform perfect interactions with humans, some limitations are inevitable when conducting HRI experiments. However, depending on the study’s purpose, researchers can leverage these limitations to an extent. In this section, we give examples of some of these limitations and insights about how to take advantage of the novelty effect, how to set expectations of the robot, and how researchers should introduce their robot to participants to adjust their expectations and limit their bias.

#### 4.3.1 Embracing the Novelty Effect

Many participants in HRI research studies have very little to no experience with robots or robotic behaviors. This leads to different, and often more positive, interactions with robots due to the newness and uniqueness of engaging with them, known as the *novelty effect*. The novelty effect is usually seen as a source of noise that researchers must mitigate, especially because different individuals experience the novelty effect differently based on their previous experience with robots, knowledge of robots, and observing robotic behavior in popular media. This prior experience creates patterns of interaction that change drastically over longer-term interactions. For example, researchers observed a decrease in interaction over time and alluded to the novelty of the robot initially causing unreasonable expectations that lead to disappointment (de Graaf et al., 2017; Kanda et al., 2004). The more limited a robot’s repertoire was, the more quickly the novelty effect fades (Leite et al., 2013). However, in certain scenarios, researchers can take advantage of the novelty effect for a positive outcome. Certain child-robot interaction studies set in a medical environment could benefit from the novelty effect through interacting with a new and unique robot or robot’s behaviors during a short visit. These interactions could reduce the fear or anxiety of a vaccination shot (Farrier et al., 2020) or even distract the child from the pain of the medical procedure (Shenoy et al., 2021; Trost et al., 2020). While these studies do not explicitly identify the role of the novelty effect, they provide a good template to observe how researchers can embrace the novelty effect to create a socially assistive robot that can generate new behaviors and distract children from painful medical procedures during each new visit. Researchers should identify similar scenarios in which their studies can benefit from embracing perceived limitations like the novelty effect.

If the research questions will likely be negatively impacted by the novelty effect, researchers should consider conducting a longitudinal study. Such studies may take place over weeks, months, or even years. Researchers should consider the pros and cons of conducting a longitudinal study. Some major benefits include understanding how people’s interaction with the robots changes over time and collecting more in-depth information. Some challenges include the time and financial commitment and vast amounts of data, which could be a benefit or challenge depending on the time a research team has to allot to the study. For researchers interested in longitudinal studies, we recommend reading about the more in-depth such as from textbooks (Menard, 2007; Bolger and Laurenceau, 2013).

#### 4.3.2 How to Leverage the Limitations and Set Expectations of the Robot

In HRI, the available set of robot functionality limits what they can do in user studies. While the current robots have extensive sensing and movement capabilities, their hardware or off-the-shelf software restricts the ability to conduct studies for prolonged durations or take them to less controlled environments (in-the-wild studies). When participants try to interact with robots that cannot appropriately respond, participants often become much more negative about them as the novelty effect fades. A straightforward solution is to set participant expectations of robot capabilities ahead of the actual study, perhaps via a familiarisation activity or pilot testing (described earlier in the paper) to determine what works best for the particular study. Two main categories of challenges are the robot’s limited interaction abilities and hardware abilities.

A common challenge in HRI is that the robots have a limited set of verbal and non-verbal reactions. This can be problematic with adults, and especially with vulnerable groups like the elderly and children, as the interaction experience becomes less naturalistic and creates misunderstandings. For example, children might find it upsetting to not have responses from a robot after performing certain tasks, which impacts their performance in the subsequent tasks. HRI researchers could incorporate a semi-structured Wizard of Oz setup to complement and augment the limited functionality of the robot, thus, enhancing the interaction experience (Riek, 2012; Steinfeld et al., 2009). Another possible solution is to run a pilot study to find the most relevant and effective tasks to be used. For instance, through piloting, researchers could identify the tasks that best complements the robot’s functionality in addition to participant behavior/response to enhance a child-robot interaction. Researchers may also wish to give children some indication of possibilities of common technical failure, like the robot running out of battery, so they are not startled if this occurs. One way to do this is to give a backstory about the robot to calibrate their expectations about the robot’s capabilities (e.g., “This is the robot’s first day out of the factory, so it is still learning to do some things”).

Hardware-related limitations also pose a significant challenge, especially in studies with prolonged durations. For instance, some robots have overheating issues when operating for an extended time and require significant down-time between uses [4]. Researchers could consider incorporating many breaks in the studies for the robot to “recover” (possibly supplemented with ice packs and keeping in cool temperatures) to enable smooth functioning over longer durations. Researchers could also employ multiple robotic platforms to identify the most appropriate setup for their experimental protocol, and enable study replicability and generalizability beyond the undertaken study.

#### 4.3.3 How to Introduce the Robot to Participants to Adjust Their Expectations and Limit Bias

As discussed previously, it is pertinent to set participant expectations of the robot’s abilities and address participants’ concerns, doubts, and inhibitions without biasing them about the experiment. It is equally vital to avoid introducing bias to participants’ interactions with the robot (Paepcke and Takayama, 2010).

Participant expectations of robot functionality and behavior depend on the robots’ appearance and the presence of other robots. If the robot’s appearance prompts participants to have excessively high expectations of the robot’s functions and abilities, the users may be disappointed after interacting with it. In contrast, if their perceptions from the interaction exceed their prior expectations, they become may more interested in the robot (Komatsu et al., 2012). Seeing multiple robots interact also affects expectations about their abilities, such as their humanlike traits (Fraune et al., 2020). Thus, researchers should consider the visual appearance and number of robots to match participant expectations or match what participants will meet in the real world.

The language and wording researchers use when introducing robots to participants also have a significant role in adjusting the participants’ expectations. When a researcher’s and robot’s instructions differ, people typically follow the experimenter’s instructions (Sembroski et al., 2017). Thus, experimenters should be careful about even subtle biases they may introduce in their instructions. For instance, a study that aims to determine the valence of a robot’s behaviors can have unwanted consequences if the researcher uses positive adjectives, like “friendly,” to describe the robot. Researchers should use impartial language and neutral adjectives to convey the functionality of robots rather than overselling or underselling them. For example, consider a study in which participants teleoperate a robot to perform a pick and place task using their motion. Researchers can avoid overpromising phrases like, “the robot can render your motion *precisely*” and under-promising phrases like, “the robot can follow your instruction *to some extent*”. One can instead say, “Control the robot using your arm motion to perform the task.”

A good way to adjust expectations while limiting bias is to have an unrelated activity or an introduction session to introduce the robot to your participant before the experiment. What this session entails would differ based on the study goals and participants. For example, if the study involves evaluating a robotic exercise coach, how researchers introduce the robot to therapists would widely differ from how they introduce it to patients. A major reason for this difference would be if therapists wish to co-design the system. In that case, they would need to understand the core functionalities of the system and its customization features to enable the system to effectively benefit the end-user. In this case, a patient might be a user of the system and would only need to know that the robot would help them perform guided exercises and that they should follow the robot’s instructions.

These introductions should cater to the attention and interest of the user. Introducing the robot to the child appropriately is crucial to foster social engagement. For example, when working with children, researchers can effectively introduce the robot through creative activities like drawing, storytelling, or theatre play. These interactions can ensure that all the children had the same background of the robot before the experiment, as having varying beliefs could affect the results (Sandygulova and O’Hare, 2018).

Depending on the study design and the robot, creating an identity for the robot can help enhance social acceptance (Duffy, 2003). Users demonstrated increased hesitancy in harming a robot with a name and a backstory compared to one without (Darling et al., 2015). Naming a robot can also heighten user familiarity with the robot (Tanaka et al., 2021). This could, in turn, increase empathy towards the robot, which may be helpful in certain studies. For example, if participants evaluate the robot’s ability to perform a pick and place task, creating an identity may not be favorable. Conversely, a study where a socially assistive robot is required to motivate users to take their medicines on time would benefit from a robot with an associated identity.

### 4.4 Collaboration

As discussed at the start of the paper, interdisciplinary and cross-team collaboration is especially important for HRI. In this section, we discuss collaborating with experts and stakeholders and co-design experiments (i.e., with people in the field), and interdisciplinary research (especially with other academic disciplines).

#### 4.4.1 Collaborating With Experts, Stakeholders, and Users in the Field to Shape the Study, and Conducting Co-design Experiments

It is essential to match solutions to real needs of the population, encouraging uptake of the eventual technology, or building confidence in the solution. In this section, we focus on designing experimental studies; this advice may not be suitable for technical papers, methods papers, or non-controlled studies. Co-design studies can be very useful for discovering user requirements, scenarios, use cases, and design guidelines by considering perspectives of the various stakeholders. Stakeholders may include *lead- or expert users* who guide the activity–like teachers, therapists, and doctors–or *end-users* such as those who would use therapy or assistance–like students, clients, and patients. Caretakers, family members, and even hospital staff can be considered stakeholders depending on how and where a robot is deployed. Collaborating with stakeholders extends researchers’ understanding of the population’s needs and the existing practices in the field. Stakeholders’ expert advice can help researchers design studies that effectively collect ground truth data, test their hypotheses, and increase the chance of technology adoption in the long term - either by developing technology to supplement existing technology or creating new technology where no technology exists. These discussions can also help researchers balance their goals with the requirements and interests of the users.

To do so, researchers can talk to experts and stakeholders to understand how their system affects users and fits into their routines. Researchers should discern the goals of the target populations, their age, their level of function, etc, and consult with stakeholders to determine appropriate robot responses. For example, researchers and stakeholders might want a robot to reinforce positive behaviors that therapists seek to instill in their clients.

One way to very closely involve stakeholders in the research process is to co-design the research with them–that is, to involve them in study design. Involving stakeholders in the design process helps ensure that the project meets their needs and desires for the technology, and it heightens their willingness to collaborate during the development and evaluation phases of the project. In these ways, co-design helps to create relevant innovations, better user experience, and improved technology acceptance. When preparing to co-design with stakeholders, researchers should carefully identify with whom they wish to collaborate, which methods to use, and how they will analyze the resulting data.

Stakeholders could also be participants in a study conducted to evaluate a robotic platform. In such studies, it is important to first accurately evaluate the target group(s) and stakeholders involved in the chosen context. Researchers may wish to study different types of stakeholders (e.g., school teachers, teaching assistants, school counselors) because they have different perspectives on the problem. However, the experiment needs to be tailored to participants based on their perspectives, so it might be best to separate groups into different co-design experiments. The research team should be clear about the desired results at the beginning of the collaboration: Do they plan to design a complete, concrete solution or first enhance the problem understanding?

There are many co-design methods to use (Šabanović, 2010). Some are more suitable for a particular target group. For adults, methods like card-sorting (Spencer and Warfel, 2004) could help participants organize their thoughts and identify relevant themes or requirements. For children, creative methods like those suggested for introducing robots, such as drawing, storytelling, and theatre play can help them express themselves. It might be difficult to go beyond the experiences that the target group is familiar with (e.g., when designing a robot, the target group may not imagine a robot able to express emotions if they think that a robot cannot emote (Neerincx et al., 2021). Creative methods can also stimulate out-of-the-box thinking. However, some participants might come up with unrealistic or less useful solutions (e.g., children designing a flying robot). Imagining someone else’s perspective can help participants in finding solutions. Including counterintuitive scenarios to evaluate with the target group might enhance creativity as well.

When creating co-design studies, researchers should consider how they will analyze the data. Because creative methods of collecting data in co-design studies often result in qualitative work (e.g., interviews, drawings of designs), researchers will most likely use textual and thematic analysis on video and audio, which can take more time than analyzing quantitative data. Analyzing the data with the research questions in mind improves clarity in the data analysis phase. However, researchers should also be alert for unexpected observations that might lead to new research questions.

#### 4.4.2 Working With Interdisciplinary Teams

One of the hallmarks of HRI research is the need to embrace an often complex and mixed-disciplinary team. Beyond the traditional problems that all interdisciplinary teams may face, including subtle differences in terminology, differing approaches and distributions of responsibilities, and differences in project management and collaboration toolsets, there are at least three commonplace but critical issues that HRI teams specifically need to address: adapting to related fields, agreeing on publication standards, and making sure all team members benefit from the collaboration.

First, while HRI researchers often adapt their analysis methods (especially statistical techniques) to those of other disciplines, the changing nature of successful methodological approaches in other disciplines means that HRI must also continue to evolve. For example, recent trends in psychology research highlight the need for empirical experiments to pre-register the study designs, sample size, and other details to ensure that researchers are not “p-hacking” (adjusting their analysis methods until a positive result is achieved; Head et al., 2015; Simmons et al., 2013) or changing their hypotheses post-hoc to confirm to the data that they have collected. To address this, HRI researchers must update their expectations and methods each year. Furthermore, researchers studying how to market robots may utilize marketing techniques, and researchers examining how individuals or groups of people interact socially with robots may use methodological techniques from social psychology (Reeves and Nass, 1997) or group dynamics literature (e.g., Fraune et al., 2019).

Second, publication standards that differ between fields can create tension and difficulties for many interdisciplinary teams. While many clinical researchers are quite satisfied to produce a solid, detailed journal paper every 18 months, computer scientists need to produce multiple high-quality (but considerably shorter) conference papers each year. As most clinical journals will not accept submissions for which the data has already appeared in another publication (regardless of field), dual submissions are also not a possibility. Research teams need to develop clear agreements on where and when publications will be submitted.

Finally, researchers from different fields are under substantially different academic pressures for advancement. While a senior clinician might be able to wait 18 months to collect data before publication, a computer science graduate student will have difficulty finding a job with such an infrequent publication schedule in unrecognized publication venues. Similarly, raising $20,000 to test a new pilot for an engineer might be a relatively easy task that results in research perks like better hardware, more students, or more frequent travel, but raising that same money will look like an impossibility to a humanities scholar (where funding is very difficult to secure) and as a critical task to a clinician (whose salary is typically not covered or only partially covered by the university). One good principle to follow is that there should be a very clear benefit to every member of a collaborative team whenever a joint project is undertaken.

### 4.5 Limitations and Future Directions

The themes that emerged during the discussions were naturally shaped by the research topics of the workshop participants (e.g., several participants worked with children with special needs). Also, some important topics did not arise during the workshop, such as the impact of COVID-19. Since this paper elaborates the results of the discussions in the workshop, some other important aspects may have been missed. Although these can be considered as limitations, we believe that one single paper could not possibly become a comprehensive guide in such a diverse field as HRI. Therefore, we hope that even with this caveat, newer members of the HRI community can find these guidelines useful and extrapolate them to their specific areas of work. In the future version of this workshop and emerging guideline, the aspects that have not been mentioned in this paper will be prioritised.

## 5 Conclusion

This paper presented the main outcomes of a workshop that aimed to connect researchers new to the field of human-robot interaction (HRI) with mentors to gain feedback on experimental designs for HRI studies. Workshop mentee and mentor feedback indicated that the workshop was very well received, with mentees stating that the feedback from the mentors did improve their study designs. Because we ran this first edition of the workshop virtually (and to account for different time zones), the sessions were short and mostly focused on small group interactions between participants and their mentors. Some aspects of improvement for future editions might include more interaction between workshop participants, for example around specific topics of common interest.

The main contribution of this paper resulted from discussions (during and after the workshop) on the main lessons learned about designing HRI studies. We reported practical guidelines organized in different themes such as study design (how to position a study regarding previous literature, how to balance exploratory vs. confirmatory research, how to define hypotheses, etc.), how to identify, recruit and work with participants in special cases (e.g., children, medical settings), how to address common limitations of HRI studies (e.g., novelty effect), as well as guidelines for successful interdisciplinary and cross-team collaborations.

## Data Availability

The raw data supporting the conclusion of this article will be made available by the authors, without undue reservation.

## Footnotes

1
https://sites.google.com/view/wysdworkshop/home

2
https://miro.com/

3 Quotes are attributed with permission
